# Single-Center Retrospective Study of Hospitalized Hepatitis A Cases in Southern Bulgaria, 2015–2023

**DOI:** 10.3390/healthcare14101428

**Published:** 2026-05-21

**Authors:** Meri Hristamyan, Simona Zlatanova, Vanya Rangelova, Ilia Tsachev

**Affiliations:** 1 Department of Epidemiology and Disaster Medicine, Faculty of Public Health, Medical University of Plovdiv, 4000 Plovdiv, Bulgaria; 2 Clinic of Infectious Diseases and Parasitology, St. George University Hospital, 4000 Plovdiv, Bulgaria; 3 Department of Infectious Diseases, Parasitology and Tropical Medicine, Faculty of Medicine, Medical University of Plovdiv, 4000 Plovdiv, Bulgaria; 4 Department of Clinical Science, Faculty of Veterinary Medicine, Medical University, 5800 Pleven, Bulgaria; 5 Department of Microbiology, Infectious and Parasitic Diseases, Faculty of Veterinary Medicine, Trakia University, 6000 Stara Zagora, Bulgaria; 6 Department of Agricultural and Forestry Sciences, Bulgarian Academy of Sciences, 1040 Sofia, Bulgaria

**Keywords:** viral hepatitis A, hospitalization, epidemiology, Bulgaria

## Abstract

Background/Objectives: The hepatitis A virus (HAV) infection continues to represent a considerable public health issue in Eastern Europe, particularly in Bulgaria, where incidence rates exceed the EU average. This study sought to investigate the epidemiological and clinical aspects of acute hepatitis A in Southern Bulgaria between 2015 and 2023 and to assess changes during the COVID-19 pandemic period. Methods: A retrospective descriptive-analytic study was conducted among 1810 hospitalized patients with confirmed acute HAV infection at a tertiary infectious diseases center from 2015 to 2023. Demographic, clinical, laboratory, and temporal data were analyzed, comparing the pre-pandemic period (2015–2019) with the pandemic phase (2020–2023). Results: Most hospitalized cases occurred during the pre-pandemic period (88.0%), with epidemic peaks observed in 2016–2017. Individuals under 18 years comprised 69.9% of cases, with a median age of 9 years and a slight male predominance of 54.9%. A notable seasonal pattern was identified, characterized by peaks in autumn and early winter. Patients hospitalized during the pandemic period were significantly older compared with the pre-pandemic period (median age 14 vs. 8 years, *p* < 0.001). Adults experienced significantly longer hospitalization and higher ALT, AST, total bilirubin, and direct bilirubin levels compared with pediatric patients (all *p* < 0.001). The median duration of hospitalization was 7 days (IQR 6–10). Two in-hospital deaths were recorded, corresponding to a case fatality rate of 0.11%. Conclusions: Hepatitis A in Southern Bulgaria mostly impacts children but exhibits changing epidemiological trends, underscoring the necessity for focused preventative methods, such as vaccination and enhanced surveillance.

## 1. Introduction

Hepatitis A virus (HAV) infection represents a major global health concern, with an estimated 100–159 million new infections annually and approximately 1.5 million clinical cases reported each year [1,2,3]. The true burden is likely much higher due to underreporting and asymptomatic infections, especially in children [4]. The age-standardized incidence rate (ASIR) of HAV has remained stable globally over the past three decades, but there is marked regional heterogeneity reflecting differences in sanitation, socioeconomic status, and vaccination coverage [5,6,7].

Bulgaria is currently classified as an intermediate-endemicity country for hepatitis A according to the WHO, with national seroprevalence data indicating that more than half of adolescents acquire immunity by age 15, but less than 90% do so by age 10 [8,9]. This level of endemicity is relatively uniform across Bulgaria but is notably higher in certain southern regions and among socioeconomically disadvantaged populations, such as Roma communities, where poor sanitation facilitates transmission [10,11]. For example, during the major 2016–2017 hepatitis A outbreak in Bulgaria, there were 35.3 cases per 100,000 population—the highest in the region—predominantly affecting children under 18 years old and linked to fecal–oral transmission in Roma communities with poor sanitation. Southern Bulgaria alone reported over 1500 hospitalizations during this period [12]. Historical surveillance data show that Bulgaria had one of the highest notification rates in Europe: 66.8 per 100,000 population in 2012 and 19.1 per 100,000 in 2018, compared to rates below 0.1 per 100,000 in Western European countries [7,13].

The COVID-19 pandemic significantly impacted hepatitis A epidemiology across Europe and in Bulgaria. Hepatitis A cases decreased significantly in all countries after COVID-19, attributed to mobility restrictions, enhanced hygiene measures, and reduced person-to-person contact [14]. For example, Romania—a neighboring country with similar epidemiological patterns—reported a decline from ~2500 cases (~12/100,000) in 2019 to just over 1000 cases (5.2/100,000) in 2020, mirroring trends seen throughout Southeast Europe and the EU/EEA region [7].

HAV infection typically begins abruptly, after an incubation period averaging about 28 days (range 15–50 days) [15]. Transmission occurs primarily via the fecal–oral route through contaminated food or water or close personal contact. While most infections are self-limiting and do not result in chronic liver disease, severe outcomes—including fulminant hepatitis—are rare (<1%) but more common among adults and those with underlying liver conditions [2,4]. In children, HAV infection is asymptomatic, with less than 10% of children under 6 years developing jaundice, and very often the only evidence of infection is the serological presence of anti-HAV antibodies [4].

Recent epidemiological shifts—driven by improvements in sanitation and incomplete vaccine coverage—have increased adult susceptibility to HAV infection in intermediate-endemicity countries like Bulgaria. This shift has led to higher hospitalization rates (32–80%) among adults compared to children and underscores the need for enhanced vaccination strategies targeting at-risk groups [7,16].

Two main types of hepatitis A vaccines exist: inactivated vaccines (HepA-I) approved in the 1990s and live attenuated versions (HepA-L). The standard regimen involves two intramuscular doses of single-antigen HepA-I, spaced 6–12 months apart, starting at age one year; combination vaccines with hepatitis B require three doses over six months for adults. Protection begins 2–4 weeks after the first dose, with boosters ensuring prolonged seropositivity [17,18]. Clinical trials demonstrate near-100% efficacy in preventing symptomatic HAV disease for pre-exposure prophylaxis, with post-exposure prophylaxis effective if given within two weeks of exposure [19,20]. The World Health Organization’s latest position paper (October 2022) recommends universal childhood vaccination in countries with intermediate endemicity or where outbreaks occur among older children/adults or cost-effectiveness analyses support it; targeted vaccination remains appropriate for high-risk groups elsewhere [9]. In Bulgaria—as in many Eastern European countries—universal childhood vaccination has not yet been implemented nationally; instead, vaccination is mainly recommended for risk groups or used reactively during outbreaks [11].

## 2. Materials and Methods

### 2.1. Study Design and Setting

This was a retrospective, descriptive-analytic case series of hospitalized patients with acute hepatitis A. The study was conducted at the Clinic of Infectious Diseases, University Hospital “St. George”, Plovdiv, Bulgaria, a tertiary referral center for the Southern Bulgaria region.

The Southern Bulgaria region has an approximate catchment population of ~1,000,000 inhabitants (2021 census estimate), covering an area of about 20,000 km^2^. The population is predominantly urban (~60%), with substantial rural municipalities and several large Roma communities (estimated 8–12% of the regional population), as well as seasonal and migrant workers. The region has moderate socioeconomic development (regional GDP per capita ~€8500, ≈80% of the Bulgarian national average), with marked intra-regional disparities in income and living conditions [21,22].

University Hospital “St. George” is the main tertiary infectious diseases referral center for this catchment area and receives the majority of hospitalized hepatitis A cases from Southern Bulgaria. However, it is not the only hospital in the region capable of admitting hepatitis A patients; therefore, the present study presents the hospitalized HAV cases at a major referral center and does not constitute a population-based incidence study.

### 2.2. Study Population and Inclusion Criteria

The study included all patients hospitalized with a diagnosis of acute viral hepatitis A between 1 January 2015 and 31 December 2023.

Acute hepatitis A infection was defined according to CDC and WHO [23,24] criteria as:

1. Clinical features consistent with acute hepatitis (e.g., acute onset of malaise, anorexia, nausea, abdominal discomfort, and/or jaundice);
2. Elevated liver enzymes (alanine aminotransferase [ALT] and/or aspartate aminotransferase [AST] > 2× upper limit of normal);
3. Laboratory confirmation of acute HAV infection by detection of anti-HAV IgM antibodies using enzyme-linked immunosorbent assay (ELISA).

Patients with total anti-HAV positivity without IgM confirmation were excluded, as total anti-HAV alone cannot distinguish acute from past infection or vaccination-induced immunity [24].

Not all individuals with laboratory-confirmed HAV are hospitalized. During the entire study period, hospitalization was generally indicated for:

- Presence of jaundice;
- ALT and/or AST ≥ 5× upper limit of normal;
- Significant systemic symptoms (prolonged vomiting, dehydration, marked asthenia);
- Underlying comorbidities (e.g., chronic liver disease, pregnancy, significant cardiopulmonary disease);
- Social/epidemiologic reasons (inability to ensure adequate care and isolation at home, crowded households, poor sanitation).

Asymptomatic or very mild cases, particularly among otherwise healthy children and young adults, were managed in outpatient settings in primary or secondary care and are not captured in this dataset.

Admission criteria were unchanged in the pre-pandemic (2015–2019) and pandemic (2020–2023) periods. During periods of high COVID-19 hospital burden, short-term triage prioritization was applied (preference for more severe cases or patients with relevant comorbidities), but the formal clinical criteria for admission for hepatitis A themselves did not change.

Demographic (age, sex), clinical (duration of hospitalization in calendar days), temporal (month and year of admission), and laboratory parameters were collected from hospital records. Laboratory data included peak values during hospitalization of serum alanine aminotransferase (ALT), aspartate aminotransferase (AST), total bilirubin, and direct bilirubin. Patients were categorized into age groups: <7 years, 8–17 years, 18–39 years, 40–59 years, and ≥60 years. Seasonal distribution was defined as winter (December–February), spring (March–May), summer (June–August), and autumn (September–November). To assess the impact of the COVID-19 pandemic on hepatitis A epidemiology, data were stratified into pre-pandemic (2015–2019) and pandemic period (2020–2023) cohorts.

### 2.3. Statistical Analysis

Statistical analysis was performed using SPSS Statistics version 24.0 (IBM Corp., Armonk, NY, USA; released 2016) at a significance level of *p* < 0.05. Continuous variables were assessed for normality using the Shapiro–Wilk test. Normally distributed variables are presented as mean ± standard deviation, while non-normally distributed variables are presented as median (interquartile range). Chi-square tests were used to compare categorical variables (gender distribution, age group proportions, and seasonal patterns) across years. For continuous variables with non-normal distribution, Mann–Whitney U test was used for two-group comparisons (e.g., male vs. female) and the Kruskal–Wallis test for multiple group comparisons (e.g., age categories).

Given the absence of a clearly defined exposure-based comparison group and the exclusive inclusion of hospitalized cases, analyses are descriptive-analytic in nature and do not estimate population-level incidence or risks.

## 3. Results

Between 1 January 2015 and 31 December 2023, 23,443 patients were hospitalized at the Clinic of Infectious Diseases. Acute viral hepatitis accounted for 2232 admissions (9.5%), of which 1810 (81.1%) were due to acute hepatitis A, representing 7.7% of all infectious-disease hospitalizations. Most hepatitis A admissions occurred before the COVID-19 pandemic (2015–2019: *n* = 1592; 88.0%), coinciding with regional outbreaks in 2016–2017, whereas only 218 cases (12.0%) were recorded during 2020–2023.

The median age of the patients diagnosed with viral hepatitis A during the study period was 9 years (5 y.o.; 25 y.o.). The youngest patient was an infant under the age of 1, while the oldest one was 79 years of age. The group of children under 18 years of age constituted 69.8% (*n* = 1263), and adults constituted 30.2% (*n* = 547) of the patients included in the study (Table 1). The age distribution of hospitalized patients differed significantly between the pre-pandemic and pandemic periods (Kruskal–Wallis H = 32.473, *p* < 0.001). Patients hospitalized during 2020–2023 were older, with a median age of 14 years (IQR 7–39), compared with 8 years (IQR 5–22) in the pre-pandemic period. We also observed statistically significant longer hospitalization among patients in the pandemic period (Kruskal–Wallis H = 6.293, *p* = 0.012).

**Figure 1:**
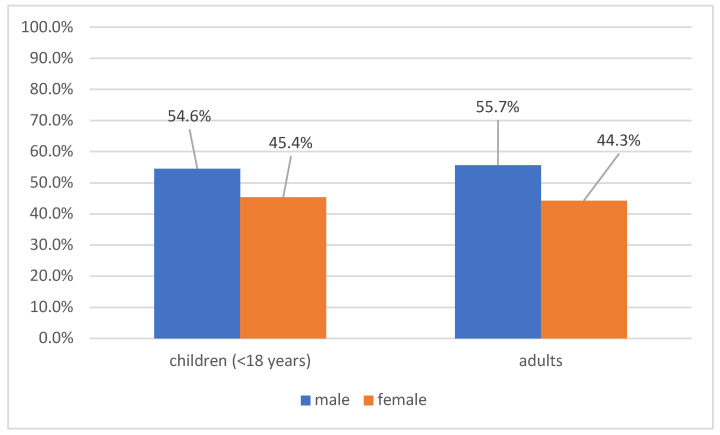
Gender distribution of patients with hepatitis A according to age (*n* = 1810).

The gender distribution of patients indicated a male predominance at 54.9% (*n* = 994). Upon additional analysis of the patient groups by age, we found that mostly men were diagnosed with viral hepatitis A in children, and a nearly identical pattern was observed in adults as well (Figure 1). There was no statistically significant difference between the gender and the age of the patients diagnosed with viral hepatitis A (ꭓ^2^ = 0.19; *p* = 0.66).

**Figure 2:**
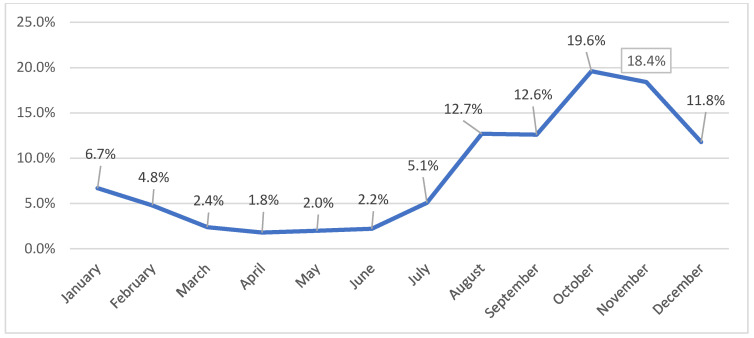
Distribution of hospitalized patients according to the month for the study period 2015–2023.

Hepatitis A admissions peaked in autumn–winter, with the highest numbers in October (354; 19.6%) and November (333; 18.4%) (Figure 2). The lowest monthly counts were observed between February and June. Travel history was not routinely collected; thus, all cases were analyzed as presumed autochthonous.

The median hospital duration during the study period was 7 days (25th percentile 6 days; 75th percentile 10 days). Patients were categorized into three groups according to duration of hospitalization. The first group included patients hospitalized for up to 7 days, comprising slightly more than half of the patients—928 (51.3%). The second group has a duration of 8 to 14 days and includes 754 patients (41.6%). The third group includes 128 patients (7.1%), for whom hospital treatment was required for more than 15 days. Adults experienced significantly longer hospitalization compared with children (χ^2^ = 261.4, *p* < 0.001) (Table 2). During the study, we explored the laboratory findings of the patients. Adults demonstrated significantly higher ALT, AST, total bilirubin, and direct bilirubin levels compared with children (all *p* < 0.001) (Table 2). Complications such as prolonged cholestasis or relapse were not systematically coded; therefore, complication rates could not be reliably estimated.

Two in-hospital deaths were recorded during the study period, corresponding to a case fatality rate of 0.11%.

## 4. Discussion

This study describes the epidemiological and clinical characteristics of 1810 hospitalized patients with acute hepatitis A treated at a tertiary referral center in Southern Bulgaria between 2015 and 2023. Most cases occurred during the pre-pandemic period, particularly during the outbreak years 2016–2017, and predominantly affected children and adolescents. These findings are consistent with the epidemiological profile of hepatitis A in Bulgaria and other intermediate-endemicity countries in Eastern Europe, where outbreaks continue to occur in populations with suboptimal sanitation [13,25].

The predominance of pediatric patients observed in the present study is comparable to previous regional data from Bulgaria, where hepatitis A outbreaks have primarily affected children living in communities with lower socioeconomic conditions and limited access to adequate sanitation [26,27,28]. However, despite the predominance of pediatric cases, adults demonstrated significantly longer hospitalization and higher laboratory markers of liver injury, including ALT, AST, total bilirubin, and direct bilirubin levels [29,30,31]. These findings suggest a more severe clinical course among adults, which is consistent with previous studies reporting that hepatitis A infection is often asymptomatic or mild in children but more clinically severe in older age groups. The median duration of hospitalization in our cohort was 7 days, although 7.1% of patients required hospitalization for more than 15 days. Prolonged hospitalization may reflect more severe hepatic involvement, cholestatic disease, or the presence of comorbid conditions [30,31,32,33].

In our cohort, 38% of hospitalized hepatitis A cases occurred in October–November, indicating an autumn–winter predominance that is compatible with fecal–oral transmission in temperate climates, where closer indoor contact facilitates person-to-person spread. Similar cold-season increases in hepatitis A notifications and outbreak activity have been reported in Europe, including during the 2016–2017 multicountry outbreak and in surveillance analyses from EU/EEA countries [34,35]. At the same time, we observed a secondary peak in August–September (25.2% of cases), suggesting that late summer and early autumn may also contribute substantially to transmission. Systematic reviews and spatio-temporal studies indicate that higher hepatitis A incidence in warmer months can be linked to behavioral and environmental factors such as travel to higher-endemicity regions, increased outdoor activities, exposure to contaminated water or uncooked seafood, and lapses in food hygiene [36,37]. Taken together, these data support a bimodal seasonal pattern in our setting, in which autumn–winter person-to-person transmission coexists with late summer and early autumn cases potentially related to travel and foodborne exposures, although individual-level risk factor information was not systematically available in our study.

A substantial reduction in hospitalized hepatitis A cases was observed during the COVID-19 pandemic period in our center. Similar declines in hepatitis A notifications have been described in multiple countries and European settings and have been linked to non-pharmaceutical interventions such as mobility restrictions, reduced international travel, enhanced hand hygiene, social distancing, and closure of schools and public venues [38,39]. In our cohort, patients hospitalized during the pandemic period were significantly older and had longer hospital stays than those admitted before the pandemic, a pattern that may reflect shifts in healthcare-seeking behavior, reduced access to routine care, and temporary triage practices during periods of high hospital burden, when admission was more likely to be reserved for patients with more severe presentations or relevant comorbidities [40,41].

Male predominance was observed both among children and adults (54.9%), although no statistically significant association between sex and age group was identified. Other regional studies from Bulgaria show comparable pediatric cohorts and male gender predominance among cases, though absolute incidence varies by area [42]. Similar male predominance has been reported in previous European outbreaks. However, detailed behavioral and exposure-related risk factors, including sexual practices, travel history, and substance use, were not systematically available in the present study, limiting assessment of specific transmission routes [43,44].

Despite the large number of hospitalized cases, mortality remained low, with only two in-hospital deaths recorded during the study period, corresponding to a case fatality rate of 0.11%. This finding is consistent with the generally favorable prognosis of hepatitis A infection, particularly in the absence of advanced chronic liver disease [45,46].

### Limitations of the Study

This study has several limitations. First, it is a retrospective single-center analysis including only hospitalized patients, which limits generalizability to the wider community and likely overrepresents clinically overt or moderate-to-severe HAV infections. Second, asymptomatic infections and cases managed entirely in outpatient settings were not captured, potentially underestimating the true incidence of HAV during the study period. Third, key contextual variables such as vaccination status, socioeconomic indicators, comorbidities, travel history, and behavioral risk factors were not systematically available in medical records, precluding detailed risk factor analysis. Fourth, complications such as prolonged cholestasis and relapsing hepatitis were not consistently coded and therefore could not be reliably characterized.

Despite these constraints, the large sample size, long observation period, and systematic inclusion of all hospitalized HAV cases at a major tertiary referral center provide robust data on the clinical severity, age distribution, and temporal trends of hepatitis A in Southern Bulgaria, offering important insights for surveillance and vaccination policy in intermediate-endemicity settings.

## 5. Conclusions

This study shows that hospitalized hepatitis A cases in Southern Bulgaria were predominantly pediatric and clustered in pronounced epidemic peaks during the pre-pandemic years, especially 2016–2017. Adults had significantly longer hospital stays and higher biochemical markers of liver injury than children, indicating a more severe clinical course in older age groups. Despite the large number of hospitalizations, in-hospital mortality remained very low, with a case fatality rate of 0.11%.

A substantial reduction in hospitalized hepatitis A cases was observed during the COVID-19 pandemic period, coinciding with public health and hygiene measures introduced during the pandemic. The continued occurrence of hospitalized HAV infections in Southern Bulgaria highlights the ongoing public health relevance of hepatitis A in the region. Strengthened preventive strategies, including targeted vaccination, improved sanitation, and continued epidemiological surveillance, remain important for hepatitis A control in Bulgaria.

## Figures and Tables

**Table 1:** Characteristics of hospitalized patients with acute hepatitis A according to study period.

Variables	Study Period 1 (2015–2019) (*n* = 1592)	Study Period 2 (2020–2023) (*n* = 218)	*p*-Value
**Age groups**			<0.001
0–6 y.o.	620 (38.9)	51 (23.4)	
7–17 y.o.	521 (32.7)	71 (32.6)	
18–39 y.o.	249 (15.6)	42 (19.3)	
40–59 y.o.	180 (11.3)	47 (21.5)	
≥60 y.o.	22 (1.5)	7 (3.2)	
**Male sex**	879 (55.2)	115 (52.8)	0.493
**Median LOS**	7 (6;10)	8 (6;11)	0.012
**LOS**			0.025
≤7 days	835 (52.4)	93 (42.7)	
8–14 days	647 (40.6)	107 (49.1)	
≥15 days	110 (7)	18 (8.2)	

**Table 2:** Comparison of length of stay and laboratory findings between pediatric and adult patients with acute hepatitis A.

Variables	Children < 18 y.o.	Adults ≥ 18 y.o	*p*-Value
**Length of Stay**			<0.001
≤7 days	795 (62.8%)	133 (24.4%)	
8–14 days	431 (34%)	323 (59.4%)	
≥15 days	39 (3.1%)	89 (16.4%)	
Alanine aminotransferase (ALT)	1286 (815;1771)	2020 (1315;3042)	<0.001
Aspartate aminotransferase (AST)	900 (468;1520)	1338 (632;2207.5)	<0.001
Total bilirubin (Tbil)	53.2 (30.8;81.9)	113.3 (76.3;160.2)	<0.001
Direct bilirubin (Dbil)	33 (15.6;46.2)	71.3 (46.2;101.5)	<0.001

## Data Availability

The data supporting the findings of this study are derived from anonymized clinical records of patients treated at University Hospital “St. George”, Plovdiv, Bulgaria. Due to ethical and legal restrictions related to patient confidentiality and institutional policies, the dataset is not publicly available. A minimal anonymized dataset (including aggregated demographic, clinical, and laboratory variables necessary to reproduce the main analyses) can be made available from the corresponding author upon reasonable request and with permission from the institutional authorities.
